# An on-chip wound healing assay fabricated by xurography for evaluation of dermal fibroblast cell migration and wound closure

**DOI:** 10.1038/s41598-020-73055-7

**Published:** 2020-10-01

**Authors:** Ghazal Shabestani Monfared, Peter Ertl, Mario Rothbauer

**Affiliations:** 1 Institute of Applied Synthetic Chemistry and Institute of Chemical Technologies and Analytics, Faculty of Technical Chemistry, Vienna University of Technology, Getreidemarkt 9/163-164, 1060 Vienna, Austria; 2 Karl Chiari Lab for Orthopaedic Biology, Department of Orthopedics and Trauma Surgery, Medical University of Vienna, Währinger Gürtel 18-20, 1090 Vienna, Austria

**Keywords:** Cell migration, Drug development

## Abstract

Dermal fibroblast cell migration is a key process in a physiological wound healing. Therefore, the analysis of cell migration is crucial for wound healing research. In this study, lab-on-a-chip technology was used to investigate the effects of basic fibroblast growth factor (bFGF), mitomycin C (MMC), MEK1/2 inhibitor (U0126) and fetal calf serum (FCS) on human dermal fibroblast cell migration. The microdevice was fabricated consisting of microchannels, pneumatic lines and pneumatically-activated actuators by xurographic rapid prototyping. In contrast to current approaches in in vitro wound healing such as scratch assays and silicone inserts in wellplate format, which show high variability and poor reproducibility, the current system aims to automate the wounding procedure at high precision and reproducibility using lab-on-a-chip. Traumatic wounding was simulated on-chip on fibroblast cell monolayers by applying air pressure on the flexible circular membrane actuator. Wound closure was monitored using light microscopy and cell migration was evaluated using image analysis. The pneumatically controlled system generates highly reproducible wound sizes compared to the conventional wound healing assay. As proof-of-principle study wound healing was investigated in the presence of several stimulatory and inhibitory substances and culture including bFGF, MMC, U0126 MEK1/2 inhibitor as well as serum starvation to demonstrate the broad applicability of the proposed miniaturized culture microsystem.

## Introduction

Mechanical trauma, burns and other diseases are attributed to the main cause of external or internal tissue damage and lesions generally known as wounds^1^. The classic stages of wound healing include inflammation, proliferation and final tissue remodeling^2^. Dermal fibroblast migration is a key step in the wound healing process. Activation of dermal fibroblasts occurs at the inflammatory stage of the wound healing process^2–4^. Cytokines and growth factors such as platelet-derived growth factor (PDGF) and interleukin-1 beta (IL-1β) that are released as inflammatory response, attract fibroblasts into the wound site^4^. Fibroblasts, originated from dermis and wound surrounding tissues, migrate along collagen matrices into the wound bed^5^. Some fibroblasts differentiate into contractile myofibroblasts^2^, that are responsible for wound contraction^6^ via alpha smooth muscle actin (α-SMA) expression^7,8^. Fibroblasts and myofibroblasts produce extracellular matrix (ECM), mainly collagen type I and III, which is necessary for cell ingrowth and wound closure^7^. Transforming growth factor β (TGF-β) and vascular endothelial growth factor (VEGF) are necessary for fibroblast cell proliferation and new tissue formation^8,9^. Apart from their role in ECM secretion, they also produce various growth factors and able to stimulate keratinocyte cell migration and proliferation. In diabetic patients, inhibition of fibroblast cell migration due to abnormal localization of EGF and FGF receptors leads to lack of stimulation of wound healing signaling cascades. Moreover, Excess in TGF-β1 level dysregulates collagen synthesis and causes the formation of unwarranted fibrosis^7^. Cell migration is an essential process in wound healing. Therefore, the analysis of cell migration is not only useful for studying the mechanisms involved in cell motility but also to study the effects of bioactive substances as therapeutic intervention of impaired wound healing and chronic wounds^9^. There are several in vitro methods that are used for cell migration analysis including scratch assays, barrier assays and microfluidic-based migration assay based on cell depletion and wounding. Moreover, various in vivo models such as excision, Incision and burn models are used for studying wound healing^10^. In excision and incision models, desired parts of healthy or damaged tissue are removed surgically to induce wounds in animals. The main applications of in vitro wound assays are: (1) analysis of collective cell migration, (2) analysis of skin cell migration for cutaneous wound closure studies, (3) discovering effects of ECM on cell migration, (4) studying the mechanism of cancer metastasis, and (5) drug screening^9^. The principle of in vitro wound healing assays is to exclude or remove cells using mechanical, enzymatic or thermal methods. After creation of a cell-free area, cell migration into the cell-free gap is monitored over several hours and days. Cell culture condition, cell seeding density and wound size are the main parameters that can affect reproducibility of in vitro wound healing assay. The most common technique in wound healing assay is known as scratch assay. Scratch assay is widely used in different fields of research such as fundamental biology, drug screening, cancer metastasis, immunology and wound healing^9^. The main tasks of this 2D assay are: (1) preparation of a cell monolayer in culture, (2) scratch the monolayer to create a cell-free area, and (3) microscopy and imaging^9^. The main advantages of this assay are being inexpensive and simple to perform. However, lack of standardization of this technique leads to low reproducibility. Manual scratch performing causes variation in wound size and quality. Therefore, this assay has usage limitations for high throughput screening in large scales^11^. The most important aspects in scratch performing is applied pressure in scratching and the angle of the scratching material. To improve the performance of wound healing assays several microfluidic approaches have been established over the years. The main principles of performing wound healing assay on-chips are to cultivate cells on microdevice platforms and creation of cell-free areas in the microfluidic channels using cell exclusion principles based on coverage of microchannel areas prior cell seeding^12^, pillar and microchannel cell retention^13,14^, enzymatic detachment of cells using laminar flow patterning^15–17^ or mechanical cell ablation using integrated pressurized actuators^18,19^.

**Figure 1:**
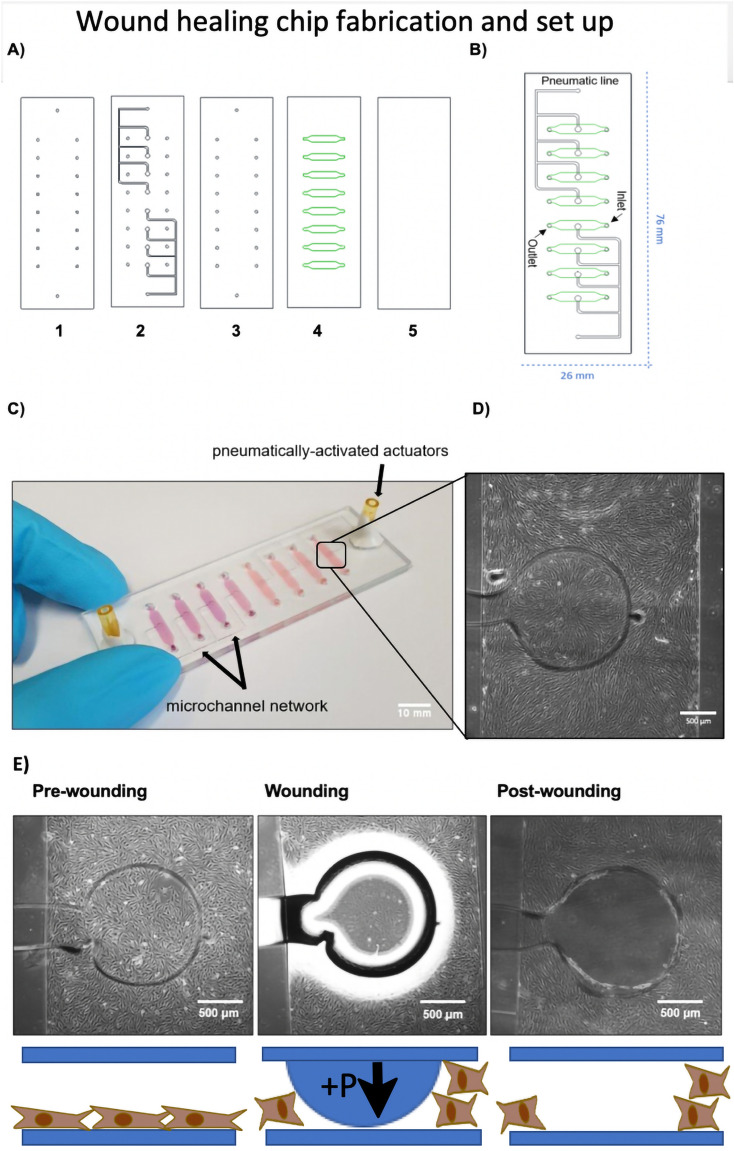
Schematic illustration of the wound healing-on-a-chip microdevice. (**A**) Structure of five different layers used in fabrication of the wound healing microdevice. (1) Drilled glass slide, (2) PDMS pneumatic layer, (3) PDMS middle layer, (4) PDMS microchannel layer and (5) glass slide. During assembly layers 1, 2 and 3 as well as layers 4 and 5 were bonded initially prior to complete assembly to ensure optimal alignment of the layers and connections. (**B**) 2D structure of the microdevice including inlets, outlets and pneumatic lines. (**C**) Actual photograph of the microdevice consisting of eight microchannels filled with pink dye featuring two individually addressable pneumatically-activated actuator ports. (Scale bar = 10 mm). (**D**) Close up view of a single microchannel with defined circular wound actuators with a diameter of 1.4 mm located in the center of the microchannels. (Scale bar = 500 µm). (**E**) Illustration of the on-chip cell depletion procedure including a pre-wounding stage for monolayer growth, a wounding stage where pressure is applied on the flexible membrane and a final analytical post-wounding stage where the cell migration of fibroblasts into the wounded cell-free area created by membrane deflection is analyzed over time. (Scale bar = 500 µm).

In the current work, the aim was to use a rapid prototyped multiplex wound healing-on-a-chip based on xurography of commercial polydimethylsiloxane (PDMS) silicone sheets as an automated, miniaturized and highly reproducible and controllable method for investigation of stimulatory and inhibitory agents and culture conditions on fibroblast cell migration and wound healing (see Fig. 1). As shown in Fig. 1A–C the multi-layered lab-on-a-chip consists of a total of five individual layers that results in 2 × 4 microfluidic channels. The microfluidic cell culture chambers are separated by micro structured pneumatic channels that are used for cell depletion using pressurized air (pneumatic layers). As shown in Fig. 1D,E the wounding protocol consists of a pre-wounding phase where human dermal fibroblasts (HDF) are grown to confluent monolayers, a wounding phase where the circular pneumatic actuator creates a wound defect in the monolayers and a final post-wounding phase that allows for fibroblast regeneration and wound closure. After initial biophysical and biological characterization, as proof-of-principle study for the application of the proposed multi-layered lab-on-a-chip device the impact of basic fibroblast growth factor (bFGF), mitomycin C (MMC), MEK1/2 inhibitor (U0126) and FCS concentrations was investigated on human dermal fibroblast cell migration and wound closure.

## Results

### Physical characterization of the rapid prototyped wound healing-on-a-chip devices

**Figure 2:**
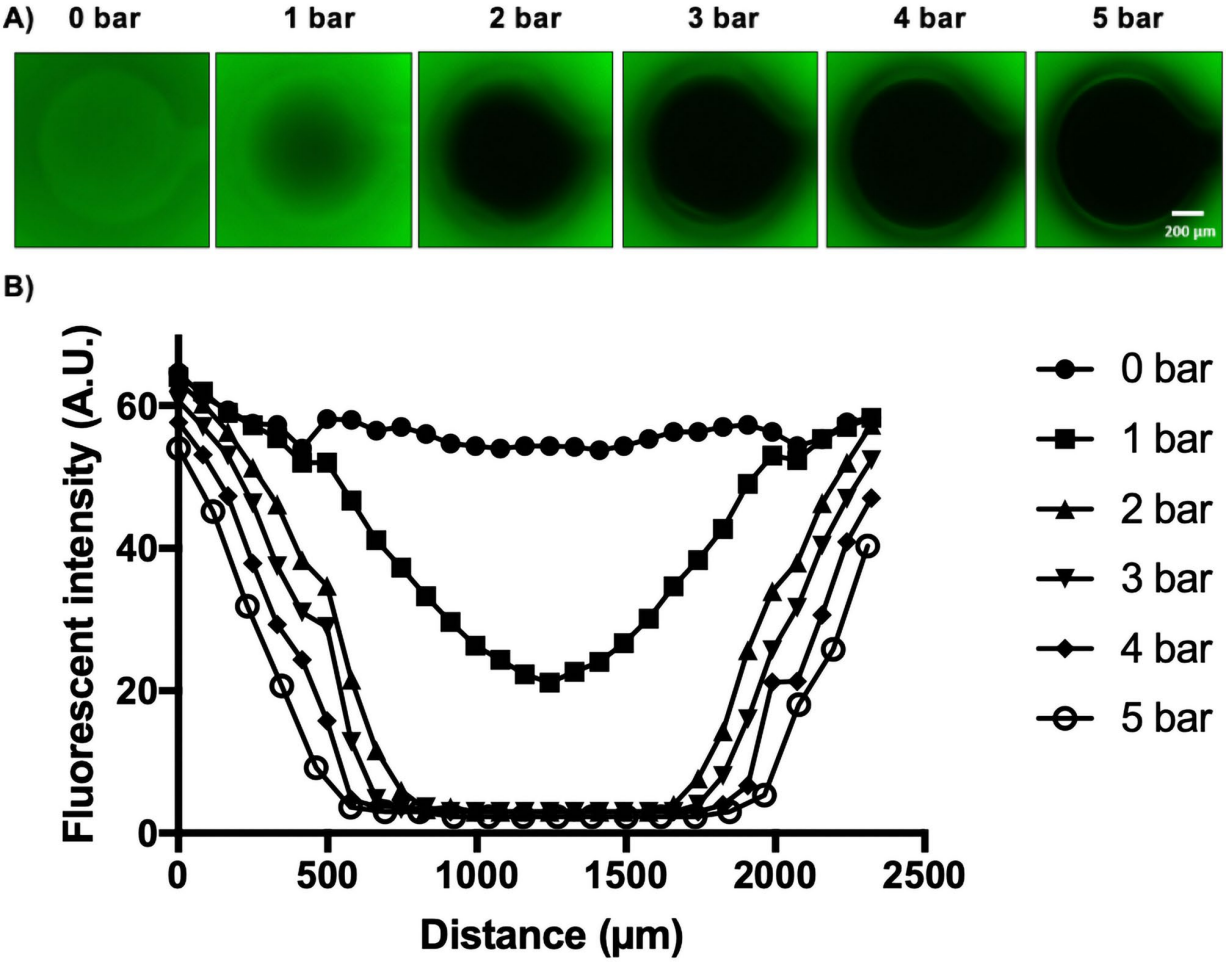
Characterization of membrane deflection and selection of optimal pressure for on-chip cell depletion. (**A**) Florescent images of a single pressurized membrane actuator at applied pressure range 0–5 bar pressure. (Scale bar = 200 µm). (**B**) Fluorescent intensity line profiles of the membrane deflection pattern in the wound area with increasing actuation pressure.

In order to analyze cell migration using the on-chip wound healing microdevices, membrane deflection and wound area was initially characterized physically. The wound healing microdevice used consisted of one top and one bottom glass slide where three PDMS layers were bonded in between forming the fluidic and pneumatic compartments. Each circular deflection area located in the center the middle of a microchannel was investigated using a fluorescent dye displacement study as shown in Fig. 2. To stimulate the membrane deflection and select the optimal pressure for cell depletion thus wound creation, the microchannels were filled with 10 μg/mL fluorescein solution and images were taken using a fluorescent microscope while application of increasing air pressure up to 5 bar applied through the pneumatic layers to stimulate the depletion method in the wounding process. (Note: higher pressure resulted in bubble formation because PDMS is gas permeable.) As shown in Fig. 2A and Video S1 fluorescent intensity in the circular wound area decreased with increasing air pressure applied to the membrane. Applying air pressure on the wounding area resulted in displacement of the fluorescein solution and change in the fluorescence intensity. The flexible membrane was deflected by applying pressure through actuators, until no change in intensity was observable. This indicated a complete removal of the underneath fluorescent liquid. A series of six representative plot lines were analyzed to illustrate the dynamics of fluorescence intensity changes in the wound area as a function of pressure thus membrane deflection in more detail. In addition, four units that were connected to a single pneumatic line were further investigated for differences in pressure thus membrane deflection, however, an overall relative standard deviation (RSD) of 5.9% was observed with the dye displacement method indicating homogenous pressure distribution along the pneumatic lines. For more detailed investigation of the optimal pressure needed to displace all fluorescent liquid thus cells in the wound area, the standard deviation of the mean fluorescence pixel intensity over the maximum cell-free area were measured (see Table 1). The reference refers to the maximum wound area that can be actuated based on the circular design constraints. This area has been used in ImageJ to analyze how the pixel intensities within the reference change when pressure is applied, and the standard deviation was calculated. The closer this deviation is to the reference value of 0.5 (non-fluorescent wound area), the lower the number of fluorescent pixels in the cell-free area. Figure 2B for instance shows that the displacement diameter between an applied pressure of 4 bar and 5 bar did not change, however, 4 bar pressure showed a higher amount of fluorescent pixel in the cell-free area. Consequently, the contact of the membrane with the culture surface is not at maximum. Therefore, 5 bar actuation pressure was chosen as optimal pressure for complete membrane deflection and thus best wound creation based on this physical characterization method in all subsequent experiments.

**Table 1:** Standard deviation analysis based on fluorescence pixel intensity inside the circular wound area of 1500 µm^2^.

Pressure (bar)	Standard deviation
0	1.98
1	7.29
2	5.37
3	2.68
4	1.79
5	0.53
Reference (no fluorescence)	0.50

### Biological characterization of the rapid prototyped on-chip wounding procedure

**Figure 3:**
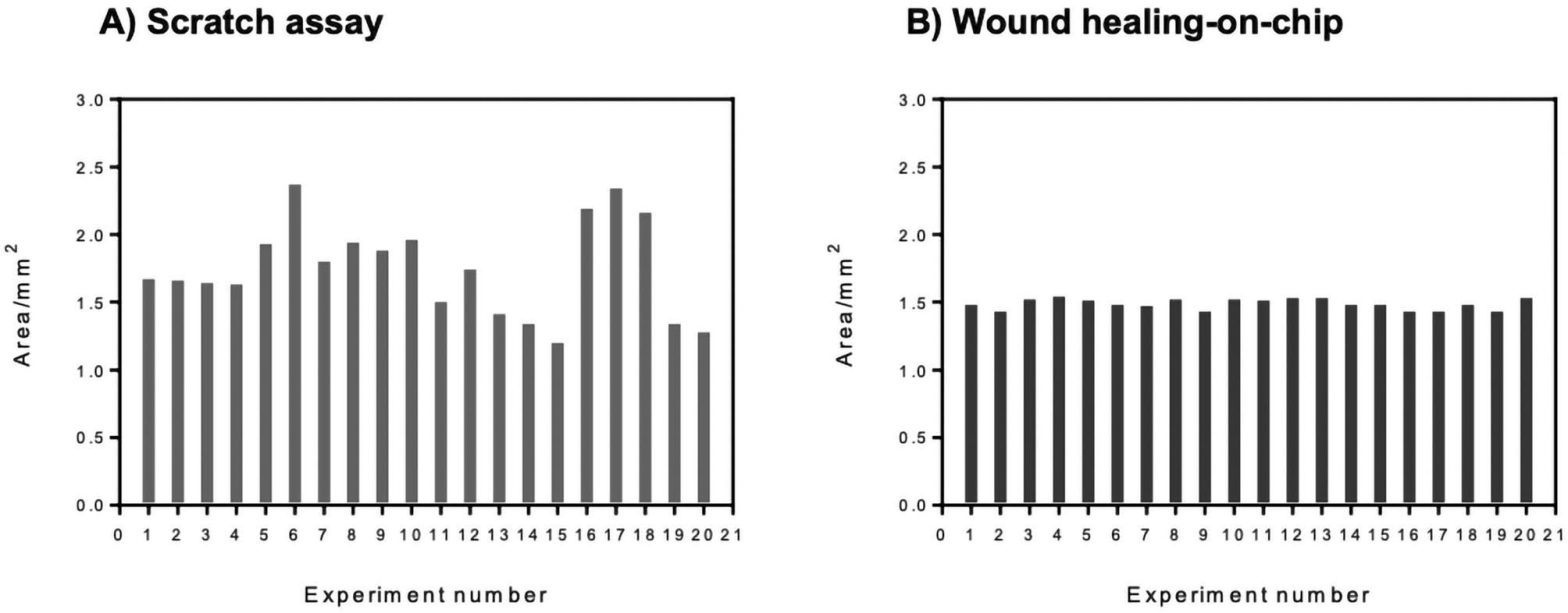
Wound area measured from scratch assay and on-chip depletion method. Each bar represents a single wound area measurement from a single experiment. (**A**) Wound areas from 20 independent scratch assay were measured. (**B**) Wound areas from 20 different experiments of on-chip depletion method.

Standard scratch assays are state-of-the-art for cell migration and wound healing studies in multi-well plates. To compare the reproducibility of wound area between the conventional scratch assay format and our on-chip wounding fabricated by rapid prototyping, 20 individual experiments were performed, and wound areas were analyzed from microscopic images using ImageJ software. The wound area variation in different experimental setups are shown in Fig. 3. The average wound area for scratch assay (Fig. 3A) was calculated to be 1.80 ± 0.35 mm^2^ from equally selected areas in each scratched well plate. (Note: Individual scratches showed hundreds of micrometers of variations within a single wound due to the manual scratching procedure with pipette tips.) In contrast, the wound healing-on-a-chip devices even though rapid prototyped using xurography, which is known to be not as precise as photolithographic microfabrication, still outperformed the scratch assay with an average wound area of 1.51 ± 0.03 mm^2^. Also, RSDs for scratch assay and the pneumatic on-chip cell depletion were 19% and 2%, respectively. Moreover, channel to channel variations accounted for 2.4% RSD for eight channels on two individually tested microdevices (n = 2). Overall, these characterizations prove that the reproducibility of wound sizes within the chip is excellent and well suited for screening with approx. 9–10-fold decrease in overall RSD compared to manual scratch assays.

Another known drawback of the scratch assay is that the manual interference will cell monolayers by a pipette tip does not only interact with the cell monolayers but also damages the underlying surface coatings and plastic surfaces. To investigate the effects of wounding on surface coating stability for both scratching and the on-chip procedure, 5 µg/µl fluorescent fibrinogen was used as surface coating and visualized by fluorescent microscopy. To compare the surface coating stability between scratch assay and on-chip wounding, fluorescent images were taken from the wound areas after wounding as shown in Fig. S1. Again, the microdevice outperformed the scratch assay. As shown in Fig. S1A pneumatic trauma at 5 bar pressure using the proposed on-chip depletion method did not interfere with the cell culture surface. This means that the surface coating remains intact after membrane deflection using 5 bar pressure loads, a pressure that has already been proven to directly contact the bottom channel surface (see also Fig. 2). In contrast, using a plastic pipette tip during scratching heavily interfered with the protein surface coating which was removed completely from the defect area (Fig. S1B).

**Figure 4:**
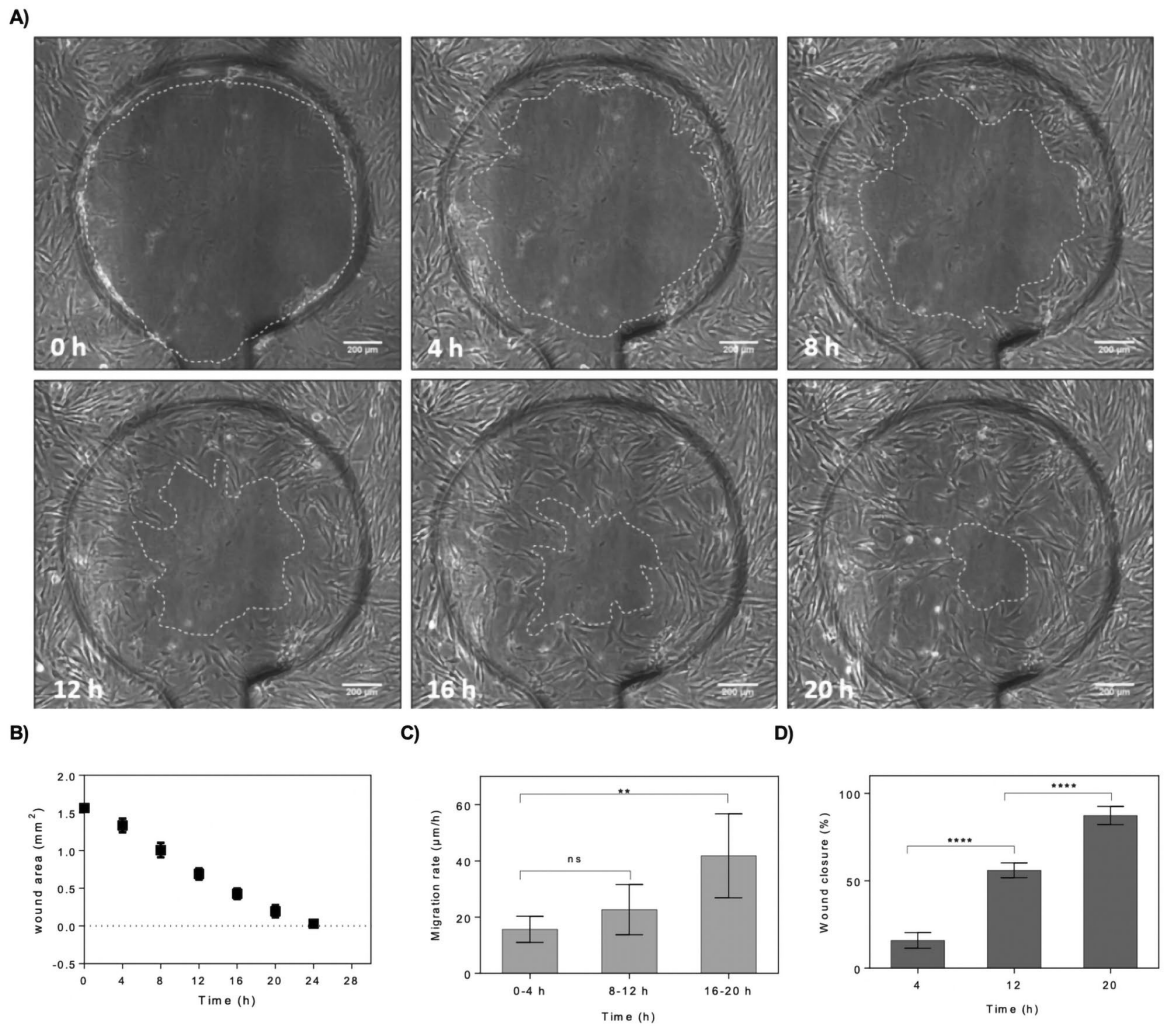
On-chip evaluation of wound closure and cell migration of wounded fibroblast monolayers maintained under standard culture conditions and complete culture medium. (**A**) Time-lapse images of the wound defect at 0, 4, 8, 12, 16 and 20 h post-wounding. The wound edges where the membrane interfaced the microchannel surface are highlighted with dashed lines. (Scale bar = 200 µm). (**B**) Analysis of average wound area as a function of cultivation time. (n = 4). (**C**,**D**) Average cell migration rate of four hour intervals for three individual time windows including 0–4 h, 8–12 h and 16–20 h post-wounding (**C**), and wound closure at three selected time-points including 4, 12 and 20 h post-wounding (**D**). Data is expressed as mean ± SD. Data sets were tested with unpaired student’s t-test with 99% confidence level, *ns* non-significant, **p < 0.01, ***p < 0.001, ****p < 0.0001.

To evaluate the impact of pneumatic on-chip cell depletion method on cell viability and cell removal efficiency, fibroblast cells were fluorescently labelled using either a calcein-AM/ethidium viability assay or Alexa Fluor-labelled phalloidin staining for visualization of f-actin. To study the wound closure and cell migration rate for HDFs, a cell monolayer was established in the microchannels and cell depletion was performed (Fig. S2A). After creation of a cell-free area using a single actuation cycle at an optimal actuation pressure of 5 bar, cell migration towards the center of the wound was observed. Lowering the pressure resulted in incomplete cell depletion that required repetitive wounding (3–4 cycles, data not shown). In general, with the present automated cell depletion method cells are forcefully removed at high precision from the confluent cell layer by pneumatic membrane deflection with a low amount of debris remaining inside the wound area. Only a small number of viable cells is pushed from the surface towards the wound edge and stays on top of the cell raft before reattachment and migration towards the center as shown in Video S2. HDF cells showed 100% viability after 16 h of wound healing. For a better visualization, fluorescent f-actin staining was performed with 50% wound closure after 16 h (Fig. S2C). After confirmation of good fibroblast viability, next wound healing parameters including wound area, wound diameter, migration rate and wound closure percentage were investigated in more detail over time for wounded HDF monolayers cultivated under standard conditions in fibroblast culture medium. Time-lapse images were taken every 4 h as shown in Fig. 4A and analyzed with ImageJ revealing that after 20 h of time-lapse imaging only a small around 200 µm sized wound defect was visible. Figure 4B shows that the average wound area measured every 4 h decreased gradually over time as a consequence of fibroblast proliferation and cell migration into the cell-free area. For instance, after a healing period of 12 h and 20 h fibroblasts already achieved around 56% and 89% wound closure, respectively. The average wound diameter (technical triplicates for n = 4) over the whole defect as shown in Fig. S3 was also measured at each selected time point and gradually decreased over time, however, manual wound diameter measurements underestimated changes in wound closure with higher standard deviation compared to automated wound closure analysis using thresholding. Also, the average cell migration rate was calculated based on the linear regression created from each average measurement over time. By dividing the slope from this line equation, the average cell migration rate during wound healing was calculated as shown in Fig. 4C. The overall average cell migration rate for HDFs was calculated to be 27 μm/h. Moreover, the cell migration within 20 h was also split in three individual time windows (0–4 h, 8–16 h and 16–20 h) to get a better understanding of migrational dynamics. The cell migration analysis showed that the migration rate and speed of cell movement increases over time from 18 µm/h in the first 4 h after wounding, 21 µm/h between 8 and 16 h and finally peaking at 40 µm/h until the end of experiment. Additionally, no significant change in the speed of migration was observed in the first 12 h of wound closure. The cell migration rate significantly increased from 8–12 h to 16–20 h time frames (N = 6, p < 0.002, difference between means = 26.13 ± 6.396). As shown in Fig. 4D, in contrast to migration rate wound closure significantly changed already during the first 12 h of healthy wound healing. This increase of cell migration speed can be attributed by the fact that the traumatic wounding by membrane deflection does not only deplete the wound area from cells but also exerts high shear from the wound edges to the lateral portions of the monolayer influencing cell–cell as well as cell–matrix interactions within the first hours of wound healing, where the fibroblasts need to reestablish thorough cell-surface interactions prior accelerating migration into the defective cell-free area.

### Effect of bFGF, MMC, U0126 and FCS on cell migration and wound closure using rapid prototyped wound healing-on-a-chip approach

**Figure 5:**
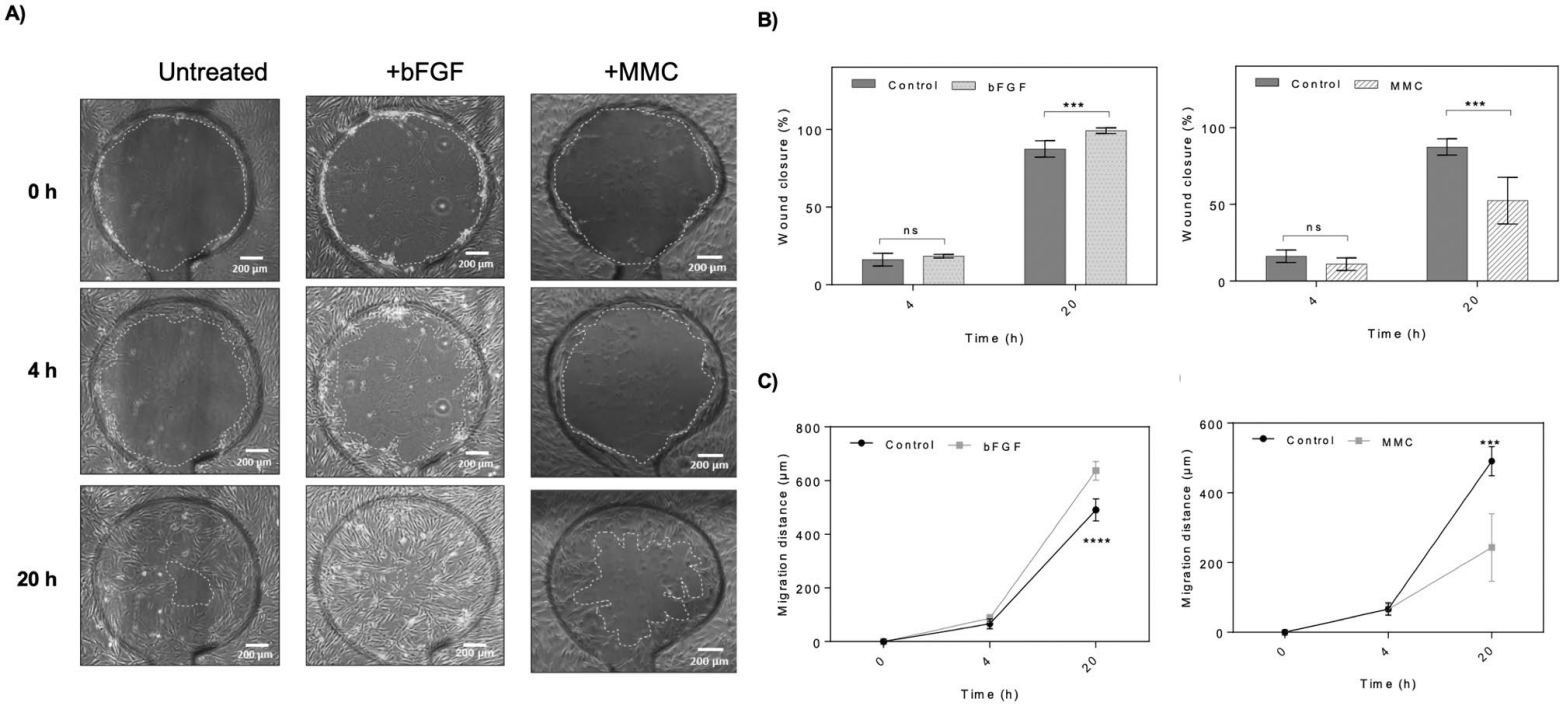
Screening of stimulatory and inhibitory effects of bFGF and Mitomycin C using the wound-healing-on-a-chip. (**A**) Representative phase contrast images of dermal fibroblast monolayers at 0, 4 and 20 h post-wounding. The wound edges are highlighted with dashed lines. (Scale bar = 200 µm). (**B**,**C**) Comparison of wound closure and migration distance of dermal fibroblast monolayers treated with 100 ng/mL bFGF or 30 µM MMC at 0, 4 and 20 h post-wounding. Data is expressed as mean ± SD. Data sets were tested with unpaired student’s t-test with 99% confidence level, *ns* non-significant, **p < 0.01, ***p < 0.001, ****p < 0.0001. (control n = 5; bFGF n = 6; MMC n = 6).

As final proof-of-principle study for screening of stimulatory or inhibitory effects of bioactive compounds on wound closure, dermal fibroblast monolayers were established inside wound-healing-on-a-chip devices. For all presented experiments, HDFs from the same batch and similar passage number (between 3 and 4 passages) were used with standard HDF growth medium as untreated control condition. After establishment of confluent, individual channels on a single device were treated or pretreated with selected compounds. Two model compounds were selected based on either stimulative or inhibitory activity on cell migration/proliferation. Basic human fibroblast growth factor (bFGF) promotes the migration of fibroblasts and stimulates collagenase synthesis was chosen as stimulant. Clinical studies reported that the level of bFGF is decreased in chronic diabetic wounds^7^, and bFGF treatment accelerates wound and improves cell migration^20^. As inhibitory agent Mitomycin C was selected because this compound is known as an anti-cancer drug that causes DNA crosslinking to prevent cell proliferation very effectively^9^ with high dosages around 300 µM arresting cell cycle in G0/G1 phase^7^. Also MMC can inhibit dermal fibroblast cell proliferation in a dose and time dependent manner^9^. Wounding was performed by application of 5 bar air pressure through the pneumatic control layers and again phase contrast images were taken from all defect sites at selected time points. A conventional scratch assay was performed prior to the on-chip wound healing assay to confirm that the selected concentrations show the expected inhibitory or stimulatory effects and no inactivation occurred during shipping and storage of the compounds. To perform such a standard scratch assay, HDF cell culture was established in a 24 well plate and the scratch assay was performed using a plastic pipette tip. Cell migration was monitored using live cell imaging in selected intervals as shown in Fig. S4. Wound area measurement and wound closure analysis as shown in Fig. S4B showed that wound closure is accelerated in presence of 100 ng/mL bFGF compared to cell to the untreated control. Pre-treatment of cells with 30 µM Mitomycin C (MMC) for 1 h prior to scratching completely inhibited fibroblast migration and wound healing. As a comparative study wound defects inside microfluidic chips were subjected to similar concentrations of MMC and bFGF after 24 h of monolayer establishment. After wounding again wound closure was monitored using live-cell microscopy as shown in Fig. 5A where bFGF improved wound healing quality whereas MMC inhibited wound closure similar to the scratch assay results. As shown in Fig. 5B,C wound area and wound diameter changes were insignificant between bFGF treated and control group at 4 h post wounding (p > 0.1). However, bFGF significantly promoted the wound closure and migration speed at 20 h compared to the control (p = 0.0009, difference between means 11.87% ± 2.457). Again, bFGF promoted the HDF cell migration and wound closure compared to the control with a difference of 11.87% ± 2.457. Also, average cell migration rate of HDFs treated with bFGF being 32 μm/h led to significant increase of the migration distance after 20 h of wound healing with relative difference of 152.2 μm ± 23.06 (p < 0.0001). Similar analysis was performed to investigate the effect of mitomycin C (MMC) on HDF cell migration and wound closure, where the fibroblast monolayers were pre-treated for 40 min with 30 µM MMC after 24 h of culture establishment. In contrast to the stimulatory effect of bFGF, MMC significantly inhibited wound closure at 20 h with difference of − 36.57% ± 6.484 wound area closure upon 40 min pretreatment with compared to the control (p < 0.001). The average cell migration rate of HDFs treated with MMC was also significantly decreased to 7 μm/h with an overall decrease in the migration distance at 20 h with a difference of − 247.8 μm ± 43.29 compared to the untreated controls (p < 0.001).

**Figure 6:**
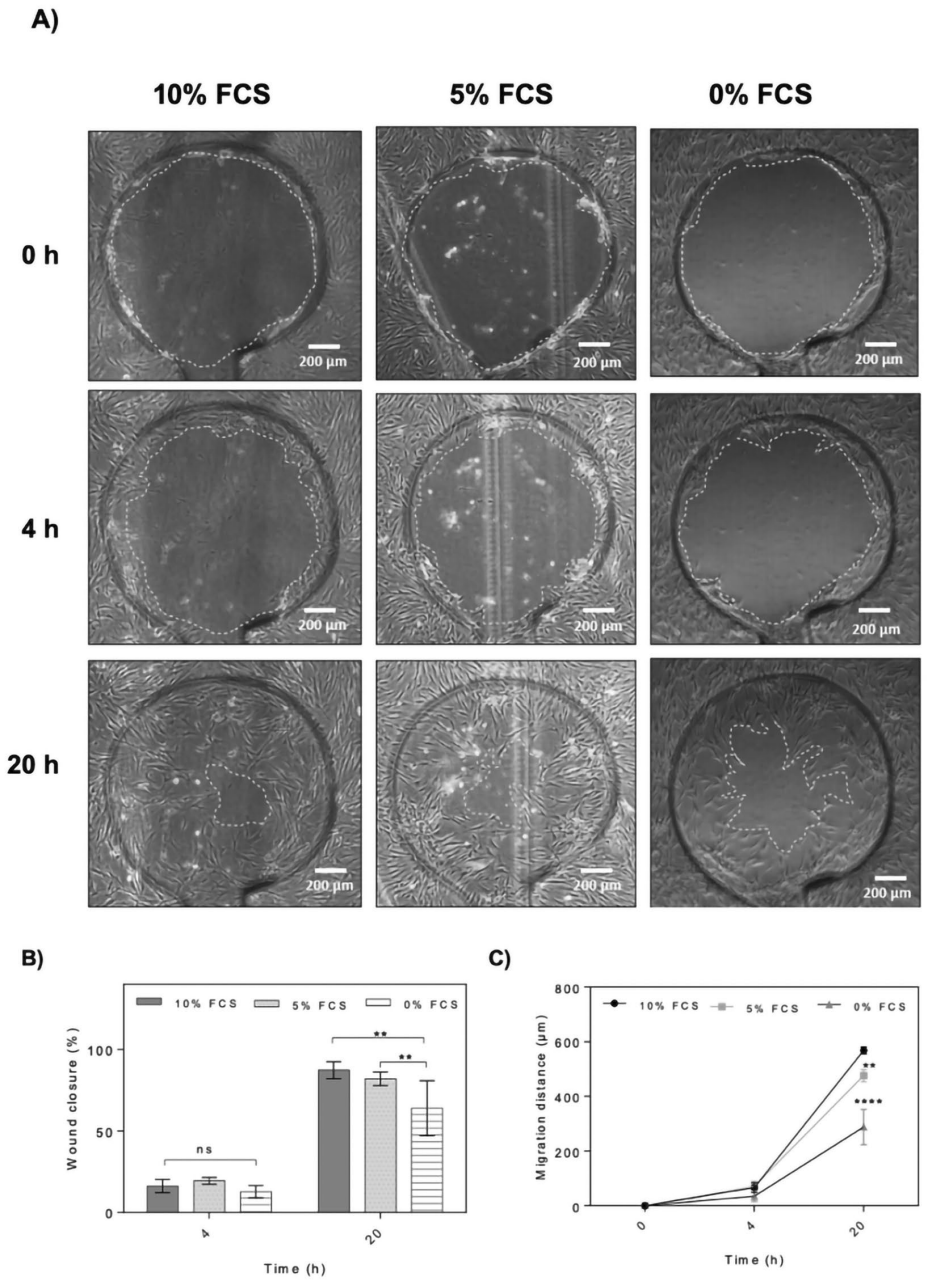
Effect of serum starvation on on-chip HDF cell migration and wound closure. (**A**) Representative phase contrast images of wound defects at 0, 4 and 20 h. The wound edges are highlighted with dashed lines. (Scale bar = 200 µm). (**B**,**C**) Comparison of wound closure and migration distance of dermal fibroblast monolayers in the presence of 10%, 5% and 0% serum supplement at 0, 4 and 20 h post-wounding. Data is expressed as mean ± SD. Data sets were tested with unpaired student’s t-test with 99% confidence level, *ns* non-significant, **p < 0.01, ***p < 0.001, ****p < 0.0001. (Control n = 5; bFGF n = 6; MMC n = 6).

Next the effect of serum depletion on HDF cell migration and wound closure on-chip was investigated because starvation is frequently used for cell cycle synchronization affects expression and production of collagen^21^ and reduces the level of expression of a-Smooth Muscle Actin (α-SMA) due to absence of growth factors such as TGF-β^9,21^. For the current on-chip study, therefore the fibroblast growth medium was exchanged with starvation medium or FCS-reduced medium containing 5% FCS (low FCS) 24 h prior to wounding. Completely supplemented HDF growth medium containing 10% FCS was used as proliferative control condition. As shown in Fig. 6 wound defect closure improved significantly in the presence of any serum condition in cell culture medium compared to the starvation group. The average wound closure after 20 h of wound healing for starvation, FCS-reduced and the untreated control samples were calculated to be 64%, 82% and 89%, respectively, with both treatment groups a significant decrease (p = 0.0014, difference between means − 29.8% ± 6.2 for starvation and p = 0.0079, difference between means − 26.1% ± 7.1 for 5% serum supplementation). Similarly, cell migration distances and speed increased proportionally with serum increase with 28, 23 and 11 μm/h for the control, FCS-reduced and starvation groups, respectively. Even though fibroblasts cultures can proliferate and migrate at reduced serum levels, cell migration is heavily affected even at 5% serum content.

**Figure 7:**
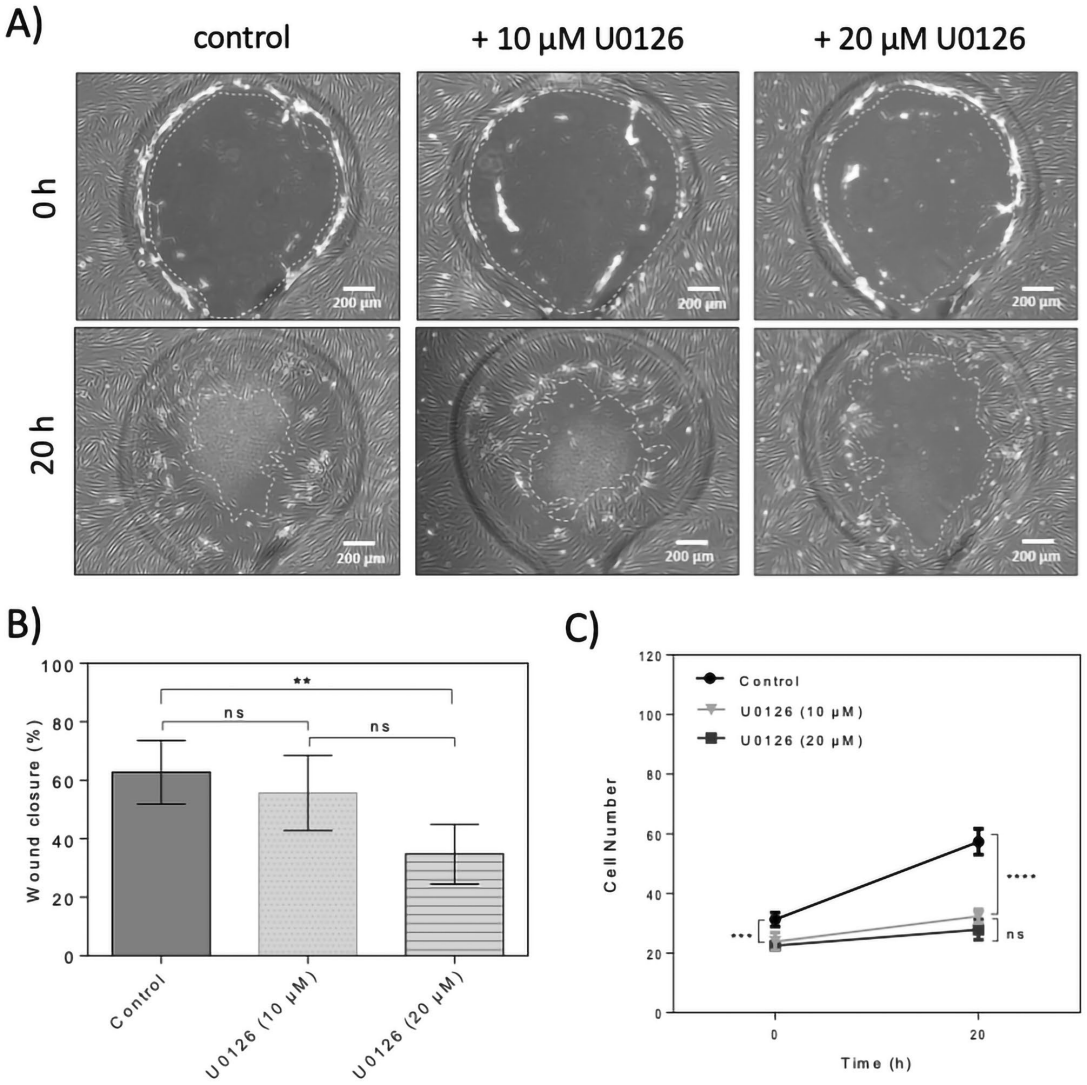
Effect of U0126 ERK inhibitor on on-chip human dermal fibroblast cell migration and wound closure. (**A**) Representative phase contrast images of wound defects up to 20 h for dermal fibroblast monolayers treated with 10 µM and 20 µM U0126. The wound edges are highlighted with dashed lines. (Scale bar = 200 µm). (**B**) Comparison of wound closure of dermal fibroblast monolayers in the presence of 10 µM and 20 µM U0126 up to 20 h post-wounding. Data is expressed as mean ± SD. Data sets were tested with unpaired student’s t-test with 99% confidence level, *ns* non-significant, **p < 0.01, ***p < 0.001, ****p < 0.0001; (control n = 6; U0126 n = 4). (**C**) Comparison of cell number in defined wound areas of dermal fibroblast monolayers in the presence of 10 and 20 µM U0126 at 0 and 20 h. Data is expressed as mean ± SD. Data sets were tested with unpaired student’s t-test with 99% confidence level, ns = non-significant, **p < 0.01, ***p < 0.001, ****p < 0.0001; (control n = 4; U0126 (10 µM) n = 3; U0126 (20 µM) n = 4).

In a final set of experiments, the role of activation of ERK signaling in cell migration was investigated. ERK phosphorylation can be inhibited using specific MEK inhibitors such as U0126^22^. ERK activation after wounding promotes keratinocyte and epithelial cell migration^9,23^, however, the ERK pathway is involved in the wound healing process through promotion of cell proliferation but not cell migration^24^. To investigate the effect of inhibition of MEK1/2 on HDF cell migration and wound closure, similarity to MMC cells were pre-treated with 10 µM U0126 for 24 h prior to wounding. After wounding using the depletion method, the wound closure was monitored using a microscope and images were taken at selected time points. Wound area measurements showed that treatment with U0126 did not have significant effect on wound closure rate compared to the control as shown in Fig. 7A,B. The average cell migration rate of HDFs treated with specific MEK1/2 inhibitor U0126 at a concentration of 10 μM was calculated to be 25 μm/h being very similar to the migration rate of 28 µm/h for untreated dermal fibroblasts which is also reflected by similar fibroblast migration distance (see Fig. S5). Therefore, the wound closure analysis in this experiment showed that treatment with U0126 at a concentration of 10 μM for 20 h did neither affect wound closure nor cell migration rate significantly by 0.11 ± 0.23 (p > 0.1; see Fig. 7B for migration rate). Increasing U0126 concentration to 20 µM significantly inhibited wound closure (p < 0.01) by almost 45%. When analyzing cell density and proliferation in more detail at the wound edges, U0126 pretreatment selectively inhibited cell proliferation (p < 0.0001) independently of the concentration as shown in Fig. 7C reducing overall initial fibroblast density from 125 ± 3 to 95 ± 3 cells/mm^2^ and 90 ± 1 cells/mm^2^ directly after wounding at 0 h for 10 µM and 20 µM U0126, respectively. Over the next 20 h of wound closure cell proliferation was inhibited by both concentrations by 26% and 32.5% of total cell count for 10 µM and 20 µM U0126.

## Discussion

To conclude a microfluidic wound-healing-on-a-chip device was successfully rapid prototyped using xurography as a miniaturized and automated platform for dermal fibroblast migration and wound closure analysis. Even though xurography does not show high fabrication resolution compared to photolithography or CNC techniques, highly reproducible circular wound areas were created on fibroblast cell monolayer by membrane deflection via pneumatic layers. To monitor cell migration, wound areas were monitored using microscopes and cell migration was evaluated by image analysis. Human dermal fibroblasts were chosen for the wound healing analysis because they are present in all wound healing phases and have a major role in the cutaneous wound healing^4^. Moreover, fibroblast cell migration is a key step in a healthy wound healing process. To analyze the chip functionality and for screening the effect of compounds on cell migration, cell depletion chip in combination with human dermal fibroblast cells were used. This study proved the functionality of the wound healing microdevice for wound healing analysis. The current comparative study demonstrated that the wound healing chip provides a highly reproducible system compared to standard wound healing assays by ten-fold reduction of RSD which indicates uniform, controllable and highly repeatable wound size creation using this automated chip-based method. In contrast to enzymatic detachment using laminar flows, the current system does not require any exchange of medium to perform wounding and can consequently be used also in the presence of biomolecules and biochemical agents. In the current proof-of-principle study both stimulatory as well as inhibitory effects of bioactive substances were successfully analyzed. Furthermore, the proposed microfluidic chips are easily integrated in automated cell culture as well as cell analysis systems and facilitate application of fluid flow and shear. For generation of flow the current system can be tilted periodically on a tilting plate to generate long-term flow needed for cell types such as endothelial cells that require shear obviating the need for tubing, syringe pumps and/or flow controllers. The current system aimed to automate the wound defect and cell depletion process completely by applying a defined pressure, whereas conventual methods require user manipulation. Integration of oxygen and impedance sensors are planned for future studies to further automate cell analysis.

## Methods

### Chip design and fabrication

The original layers structure was published previously^19^ and adapted to hold eight individual culture chambers with two addressable pneumatic lines. The wound healing microdevice consists of three PDMS layers: a top layer with pneumatic structure, a middle plane layer and bottom layer with microchannel networks. The original design consisted eight pneumatic ports which was changed and reduced to two ports. The design was adapted as each pneumatic port was connected to four wounding areas. With this design simultaneous wounding was performed for four channels, with each time pressure application through each pneumatic port. PDMS foil with 250 µm thickness (MVQ Silicones GmbH, Germany) was cut into 76 × 26 mm designed structures using a cutting plotter machine using xurography as previously optimized^9,25^. After removal of one protection plastic layer from PDMS foils, the foils were placed on cutting stage, position and size of the structures were adjusted using CutStudio software and the structures were cut to specific designed patterns. Two microscope glass slides were used on top and bottom of the microdevice. Structures were drilled on the top glass slides using a drill press machine. Drill needles with a head of 1 mm diameter were used to drill the holes. The glass slides were further cleaned using ultrasound bath in three steps with 2% Hellmanex III solution, isopropanol and DI water. After cleaning, the slides were air dried and kept in a 70 °C oven to dry completely. The glass slides and PDMS foils were bind to each other using a plasma cleaner machine. Each two layers were treated with plasma for surface activation and then carefully aligned on top of each other. After binding, the layers were pressed and baked for one hour at 70 °C in the oven. First, the pneumatic layers (layer 2 and 3) were bonded to the top glass slide (layer 1) and the microchannel (layer 4) to the bottom glass slide (layer 5). After one hour baking, the glass-pneumatic structures were bind to the middle PDMS membranes and baked for one hour at 70 °C in the oven. In the last step, the glass-pneumatic-middle layer were bind to the glass-microchannel layers. The microdevice consists of eight microchannels with 2.5 mm width, 8.5 mm length 0.25 mm height. The volume of each microchannel was estimated to be 7 µL. The surface area of each microchannel was calculated to be 26.5 mm^2^ and each defined circular wound area was estimated to be 1.5 mm^2^. After building the microdevice, polymer tubes were glued to the ports on top of the glass slides using epoxy glue. Additionally, by optimizing the cell cultivation conditions on chip, the polymer ports were replaced by PDMS reservoirs. To cast reservoirs, silicon curing agent and silicon elastomer base were mixed with 1:10 ratio. After degassing the silicon mixture using a vacuum pump, the mixture was poured into reservoir forms and baked for one hour at 70 °C. A 6 mm biopsy puncher was used to remove the reservoirs structures. Reservoirs were bind to the top of the microdevice using plasma technology and baked for one hour at 70 °C in the oven.

### Chip characterization (actuation and surface coating stability)

To select a suitable pressure for wounding and stimulating the membrane deflection, microchannels were filled with 10 μg/mL fluorescein solution in DI water. Then a pneumatic pump was connected to pneumatic activated actuators and by opening the valve, air pressure was applied on the membrane. 1–5 bar pressure were applied to the membrane respectively and pictures were taken at each step using the fluorescent microscope. Images were taken while the membrane was deflected under 1–5 bar of air pressure. Further image analysis of the fluorescent intensity was done using ImageJ software by transforming images to 8-bit gray scale, combination into a stack and thresholding (min 0 to max 28, dark background selected) prior to deflection area calculation (measure function). For generation of line profiles, a linear region of interest was placed on a stacked image and plot profiles were analyzed (Plot Profile function).

To evaluate the stability of the surface coating, a well from a 48 well plate and a microchannel on the wound healing chip were coated with fluorescent conjugated fibrinogen for 4 h. Mechanical wounding was performed on chip by the depletion method via pressure application and the scratched assay was performed using a plastic pipette tip. Images were taken using a fluorescent microscope before and after wounding for comparative studies.

### Cell culture and bioactive compound preparation

Pooled primary human dermal fibroblasts derived from juvenile foreskin (HDFp.05.C; lot# EB1104281; > 3 donors) were purchased from a commercial supplier (CELLnTEK).Cells were weekly split enzymatically and maintained incubated at 37 °C and 5% CO_2_ using either complete growth medium (DMEM high glucose, 10% FCS, 1% Antibiotics; Gibco), starvation medium (DMEM high glucose, 1% Antibiotics) or FCs-reduced growth medium (DMEM high glucose, 5% FCS, 1% Antibiotics). All methods were carried out in accordance with internal ethical as well as safety and scientific guidelines and regulations on working with commercial human cell lines. Bioactive compounds including bFGF, U0126 and mitomycin C stocks (Sigma Aldrich—Merck) were prepared according to the manufacturers’ description. After preparation of stock solutions, they were further diluted in HDF growth medium to reach the desired concentration. In brief, U0126 was prepared as 1 mM DMSO stock diluted to 10 μM working solution. Mitomycin C was prepared as 1 mM stock in deionized H_2_O and diluted to a final concentration of 30 μM. bFGF was adjusted to 5 μg/mL in Tris buffer and diluted to 100 ng/mL working solution.

For on-chip wound healing experiments, prior to surface coating, the microchannels were rinsed twice with 70% EtOH and DPBS respectively. The microchannels were filled with 0.09% collagen I solution (collagen type I in DPBS) and incubated for 2 h at 37 °C. HDF cells were washed twice with DPBS and treated with trypsin for 3–5 min until detached. The HDF growth medium was added to the cells and the suspension was centrifuged at 170 rcf for 5 min. The cell pellet was resuspended in the fresh medium and cell number was determined. The cell concentration was adjusted to 600 cells/μL. After removing the coating solution, the microchannels were filled with the cell suspension until the reservoirs were filled to the top. The chips were then incubated at 37 °C, 5% CO_2_ for cell adhesion. The next day medium was changed to HDF growth medium, starvation medium, FCS-reduced medium or medium containing hβFGF. For screening the effect of compounds on cell migration, HDF cells were treated with 30 μM mitomycin C for 40 min or U0126 10 μM for 24 h, prior to wounding. After medium change or pre-conditioning, the reservoirs were sealed with PCR plate tapes to prevent medium vaporization and contamination. To have a consistent comparison between different conditions, HDF cells from comparable passage were used. For pneumatic wounding, 72 h after seeding, wounding on chip was performed using cell depletion method. The pressure of the pneumatic system was set to five bars. Then pneumatic pump was connected to pneumatic activated actuators using a plastic connector. By opening the valve, air pressure was applied on the flexible membrane which was pressed on the cell monolayer. Circular cell-free areas were induced mechanically within 10 s of membrane compression. After wounding, the pneumatic pump was disconnected from the pneumatic activated actuators.

### Scratch assay

To prepare samples for the scratch assay, wells from 24 well plates were coated with 0.1% gelatin solution (gelatin in DI water) and incubated for 2 h at 37 °C. After removing the coating solution HDF cells were seeded with cell density of 5000 cells/cm^2^ on the well plate. Well plates were incubated at 37 °C, 5% CO_2_ until a confluent monolayer of cells in each well is formed. The medium was changed to HDF growth medium, medium containing hβFGF the next day or cells were treated with 30 μM mitomycin C prior to scratching. 72 h after seeding, scratch assay was performed by manual scratching. Cell-free areas were created by mechanical removal of the cells from the cell monolayer, using a plastic pipette tip.

### Live-dead and phalloidin staining

To evaluate viability after wounding or during wound healing, the cells were stained with calcein AM and Propidium iodide (PI) with the volume ratio of 1:1000 in basal DMEM medium. The cells were incubated with the staining solution at 37 °C, 5% CO_2_ for 40 min. After staining, the cells were washed twice with DPBS and covered with basal DMEM medium for fluorescent imaging. By choice, phalloidin and Hoechst 33342 staining was performed 24 h after wounding as an end point staining. Cells were washed twice with DPBS and incubated with Triton X-100 fixation solution for 10 min. Then, the cells were stained with phalloidin and Hoechst with the volume ratio of 1:1000 and 1:2000, respectively. The cells were incubated with the staining solution in DPBS for 30 min which was followed by fluorescent imaging.

### Microscopy

After wounding, the wound closure was monitored using live cell microscope incubation system (CellVivo) or manually using a live-cell cell culture microscope IX81 (Olympus). Samples were positioned on the microscope stage and the light intensity was adjusted. 4× magnification objective was selected for observation of the wounded area. Coarse and fine focus knobs were used to bring the specimen into optimal focus. The brightness of the image was adjusted, and pictures were taken in phase contrast mode. Live cell incubation system and microscope was used for time lapse and fluorescence imaging. The sample was positioned on the microscopic stage. The sample was covered with the cover plate connected to the CO_2_ supply. Substitutivity, medium containing 10% HEPES was used. Temperature and CO_2_ level were adjusted to 37 °C and 5% using the environment control chamber. The image setting was set to the phase contrast mode and imaging positions were selected manually. The specimen was brought into focus using focus knobs. After position confirmation and focus adjustment, the desired interval between time lapse imaging and number of imaging cycles were selected in the image acquisition section. By pressing start key, time lapse imagining with defined intervals was started. For fluorescent imaging, first the specimen was brought into focus using focus knobs. The setting was changed to fluorescent mode with multichannel imaging techniques depending on fluorescent dyes color. DAPI, FITC and/or TRITC channels were selected, and fluorescent intensity was adjusted by changing the exposure time.

### Image analysis of cell-free areas using ImageJ software

To analyze the images taken by microscopes, the pictures were exported in JPG format. After opening the images in Image J software, the unit for distance measurement was changed from pixel to micrometer. This change of the scale was applied to all images prior to image analysis by drawing a free line over the scale bar and selecting the “Set Scale” key into desired unit.

To measure the cell-free area, the wound edges were cleared using “Find Edges” and “Sharpen” keys. The color threshold of images was also adjusted to “black and white” format using “Image Adjust” key. After threshold selection, the wound edges and cell covered areas appeared in white color and the cell-free area appeared in black. By selecting “Analyze Particle” key, the percentage of wound area was measured. Substitutivity, the wound area was selected manually using “Polygon selection” from the task bar. Additionally, wound diameters were measured by using the “Freehand selection” tool, after scale setting and color threshold adjustment for three lines per wound sample. The measurement results were exported as Excel files for further analysis. The established formulas are used for cell migration and wound closure quantification. The wound width was measured at desired timepoints and average widths were calculated. By subtraction of final wound width W(t_2_) from initial wound width W(t_1_), the change in wound width between selected timepoints was calculated. Additionally, by division of this number by the duration of cell migration ($\Delta t$). This number was then divided by 2, since the cell migration occurs from both wound edges as shown in Eq. (1).

1 $$ Migration\;rate \left( {{\raise0.7ex\hbox{${um}$} \!\mathord{\left/ {\vphantom {{um} h}}\right.\kern-\nulldelimiterspace} \!\lower0.7ex\hbox{$h$}}} \right) = \frac{{W\left( {t1} \right) - W\left( {t2} \right)}}{2*\Delta t} $$

The wound areas were measured, and the average wound size was calculated at desired time points. Final wound area A(t) was subtracted from initial wound area A(t_0_) and this number was divided by the initial wound area as shown in Eq. (2).

2 $$ Wound\;closure\;\left( \% \right) = \frac{{A \left( {t0} \right) - A \left( t \right)}}{{A \left( {t0} \right)}} \times 100 $$

To quantify the wound size reproducibility and be able to compare the wound size reproducibility between conventional scratch assay and wound healing assay-on-chip, wound areas were measured from 20 wounds. From 20 single measurements, the average wound size (X̄) and standard deviation (SD) were calculated. The relative standard deviation was reported in a percentage value for each series of experiments as shown in Eq. (3).

3 $$ RSD\;\left( \% \right) = \frac{SD}{X} \times 100 $$

### Statistical analysis

For statistical analysis, data sets were tested for significance using GraphPad Prism software (Version 6.0). The data are presented as the mean ± standard deviation (SD). Unpaired student’s t-test with confidence interval of 99% used for analysis of differences between groups. Two-tailed p values were calculated, and differences were considered significant at p < 0.01. For unbiased automated image analysis of wound closure, the thresholding tool of ImageJ was used prior to analysis of wound closure and cell migration.

## Supplementary information

Supplementary Figures.

Supplementary Video 1.

Supplementary Video 2.

## Data Availability

Data is available upon email request.
