# Supplementary material for: An on-chip wound healing assay fabricated by xurography for evaluation of dermal fibroblast cell migration and wound closure
Source: Sci Rep. 2020 Oct 1;10:16192. doi: 10.1038/s41598-020-73055-7 (PMC7529912; doi:10.1038/s41598-020-73055-7)
Supplement: Supplementary file 1 — Supplementary Figures. [file 41598_2020_73055_MOESM1_ESM.docx]

**Supporting information: An on-chip wound healing assay fabricated by xurography for evaluation of dermal fibroblast cell migration and wound closure**

G. Shabestani Monfared ^1,^ *^⊥^*, P. Ertl ^1,*^ and M. Rothbauer^1,2,^ *^⊥,^*^*^

*^1^ Institute of Applied Synthetic Chemistry and Institute of Chemical Technologies and Analytics, Faculty of Technical Chemistry, Vienna University of Technology, Getreidemarkt 9/163-164, 1060 Vienna, Austria*

*^2^ Karl Chiari Lab for Orthopaedic Biology, Department of Orthopedics and Trauma Surgery, Medical University of Vienna, Währinger Gürtel 18-22, 1090 Vienna, Austria*

*^⊥^ these authors contributed equally*

~~
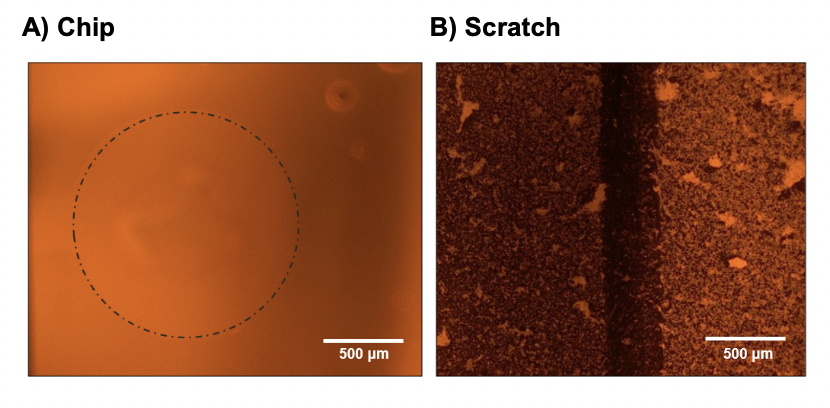
~~

**Fig. S1** Impact of cell depletion method on surface coating. Fluorescent images of culture surfaces coated with fluorescent fibrinogen after wounding using (A) on-chip depletion method at 5 bar pressure and (B) standard scratch assay with disposable plastic pipette tips. The circle represents the applied wound area. (Scale bars = 500 µm)

~~
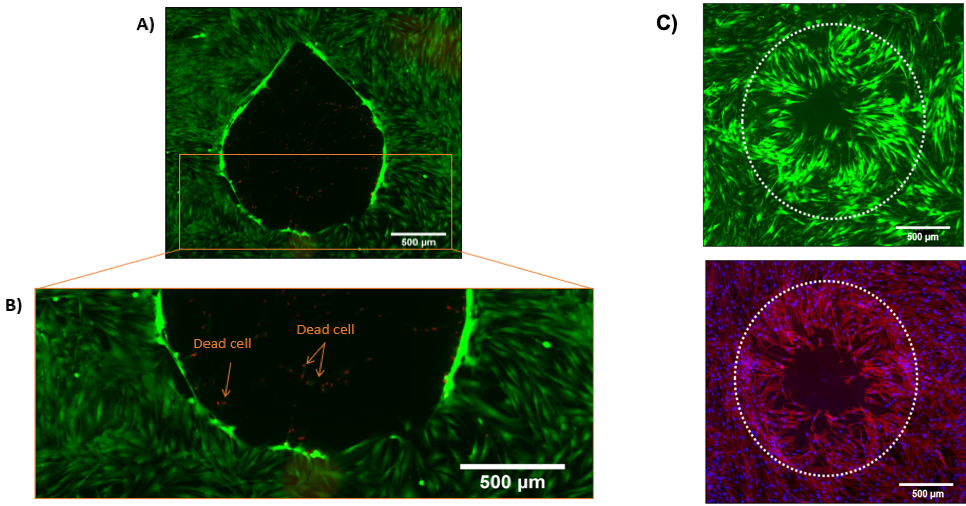
~~

**Fig. S2** Live-dead staining of HDFs after wounding. (A) HDFs were stained immediately after wounding with calcein AM-PI. (B) Cytoplasm of live cells were stained with calcein AM (green) and DNA of dead or damaged cells interact with PI (red). (Scale bar = 500 µm) (C) Live-dead staining (top) and phalloidin/DAPI staining (bottom) with nuclei (blue) and actin filaments (F-actin; red) 16 hours post wounding. The dashed line represents the initial wound area. (Scale bar = 500 µm)


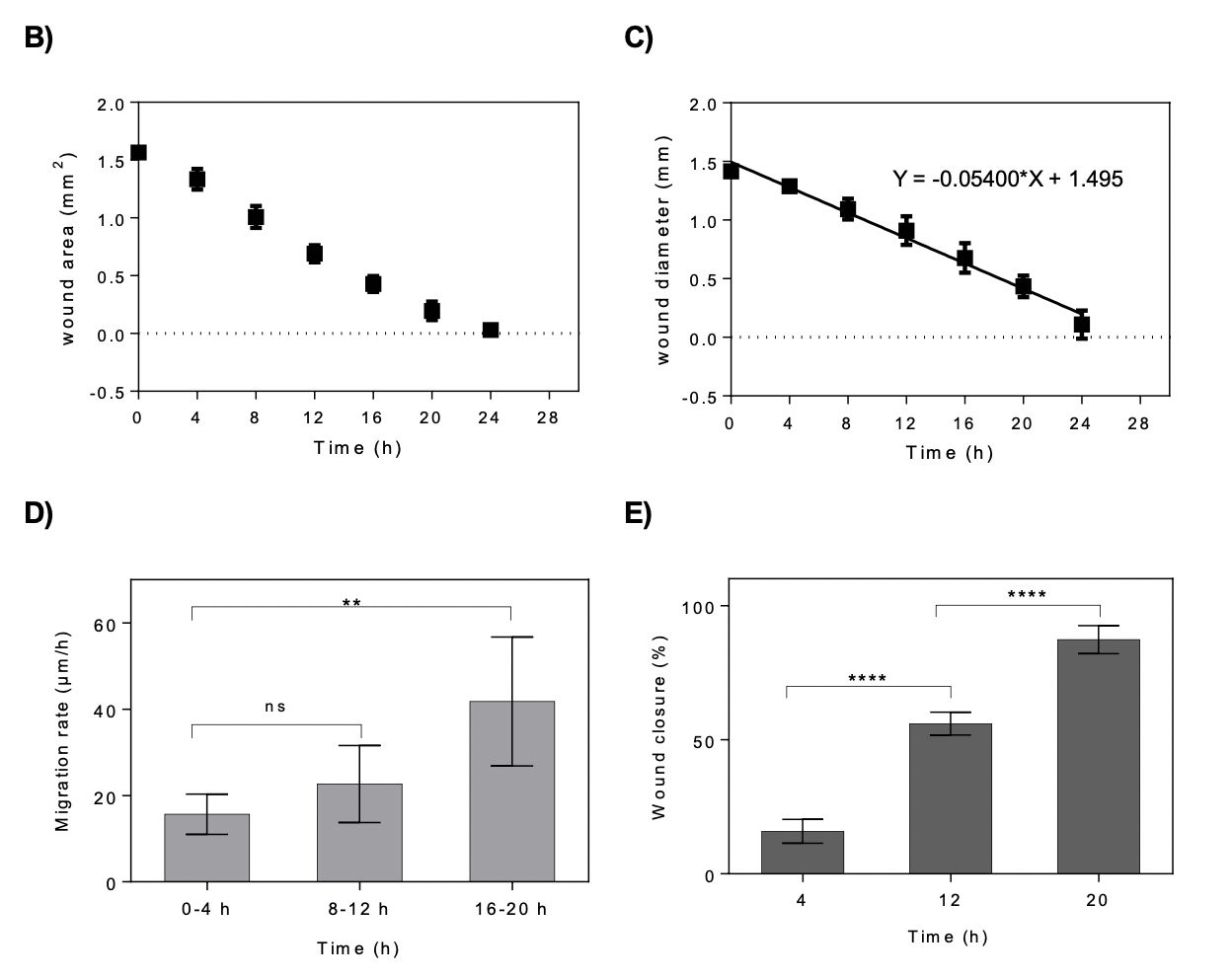


**Fig. S3** Average wound diameter and linear regression for manual analysis of wound healing (R^2^ = 0.95; n=4).


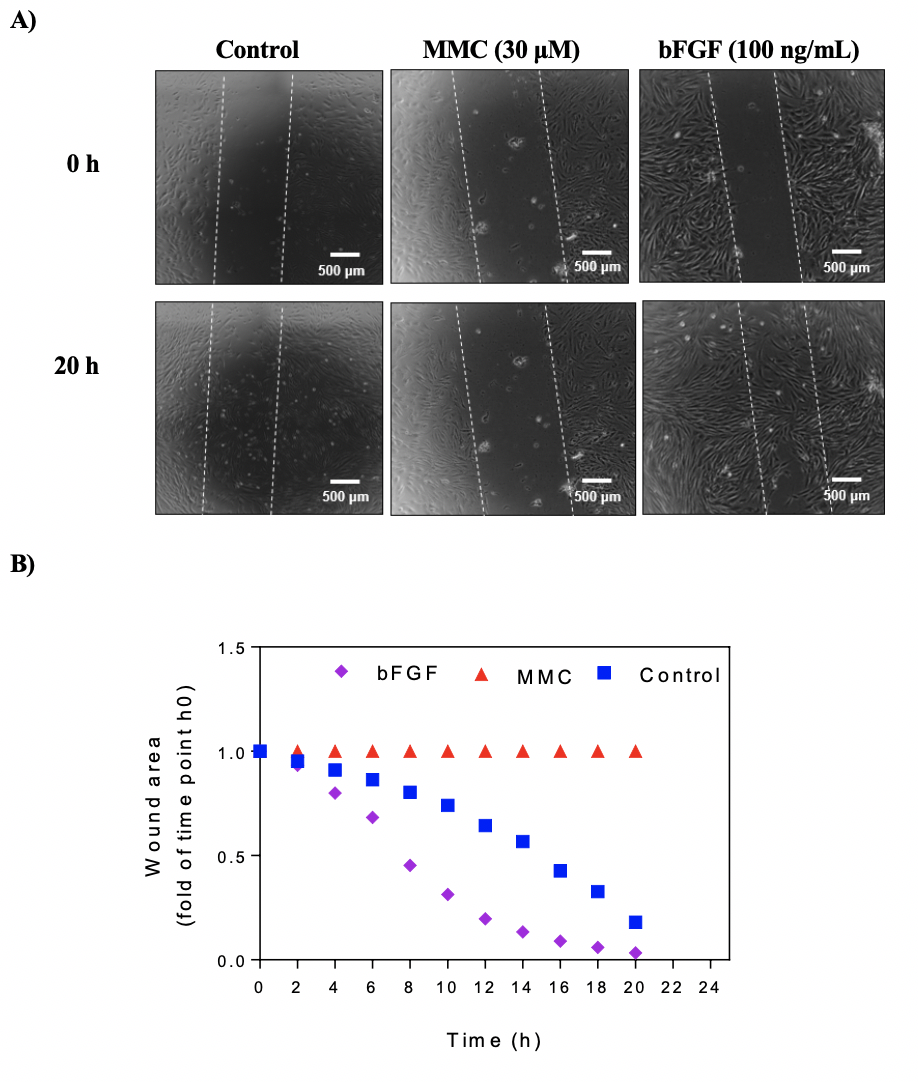


**Fig. S4.** HDF cell migration assay (scratch assay). (A) Images show wound closure visualization at 0 and 20 h post-scratch (Scale bar = 500 µm). (B) The graph shows the comparison of the wound closure in 3 groups. Wound area analysis shows that cell migration in the group treated with MMC (30 μM) for 1 hour is inhibited. The wound area in the group treated with bFGF (100 ng/mL) is significantly decreased compared to the control group (P <0.0001). (N=3, mean ± SD)

**Fig. S5**. Impact of U0126 ERK inhibitor at 10 µM on HDF cell migration distance (control n=6; U0126 n=4). Data is expressed as mean ± SD.

**Contributions**

G.S.M. and M.R. contributed equally to this work. G.S.M. and M.R conceived the experiments, performed the experiments. G.S.M. analyzed the data; G.S.M. prepared the figures; G.S.M., M.R. and P.E. wrote the manuscript.

**Competing Interests**

The authors declare no competing interests.

**Data Availability**

Data is available upon email request.

**Corresponding author**

Correspondence to Mario Rothbauer (mario.rothbauer@meduniwien.ac.at) and Peter Ertl (peter.ertl@tuwien.ac.at)
